# CD98 heavy chain as a prognostic biomarker and target for cancer treatment

**DOI:** 10.3389/fonc.2023.1251100

**Published:** 2023-09-26

**Authors:** Pu Xia, Anna Dubrovska

**Affiliations:** ^1^ OncoRay - National Center for Radiation Research in Oncology, Faculty of Medicine and University Hospital Carl Gustav Carus, Technische Universität Dresden and Helmholtz-Zentrum Dresden-Rossendorf, Dresden, Germany; ^2^ Helmholtz-Zentrum Dresden-Rossendorf, Institute of Radiooncology-OncoRay, Dresden, Germany; ^3^ German Cancer Consortium (DKTK), Partner Site Dresden and German Cancer Research Center (DKFZ), Heidelberg, Germany; ^4^ National Center for Tumor Diseases (NCT), Partner Site Dresden: German Cancer Research Center (DKFZ), Heidelberg, Faculty of Medicine and University Hospital Carl Gustav Carus, Technische Universität Dresden, Helmholtz-Zentrum Dresden-Rossendorf (HZDR), Dresden, Germany

**Keywords:** CSC: cancer stem cell, CD98hc, therapy resistance, tumor progression, LAT1, xCT, ferroptosis

## Abstract

The SLC3A2 gene encodes for a cell-surface transmembrane protein CD98hc (4F2). CD98hc serves as a chaperone for LAT1 (SLC7A5), LAT2 (SLC7A8), y^+^LAT1 (SLC7A7), y^+^LAT2 (SLC7A6), xCT (SLC7A11) and Asc1 (SLC7A10) providing their recruitment to the plasma membrane. Together with the light subunits, it constitutes heterodimeric transmembrane amino acid transporters. CD98hc interacts with other surface molecules, such as extracellular matrix metalloproteinase inducer CD147 (EMMPRIN) and adhesion receptors integrins, and regulates glucose uptake. In this way, CD98hc connects the signaling pathways sustaining cell proliferation and migration, biosynthesis and antioxidant defense, energy production, and stem cell properties. This multifaceted role makes CD98hc one of the critical regulators of tumor growth, therapy resistance, and metastases. Indeed, the high expression levels of CD98hc were confirmed in various tumor tissues, including head and neck squamous cell carcinoma, glioblastoma, colon adenocarcinoma, pancreatic ductal adenocarcinoma, and others. A high expression of CD98hc has been linked to clinical prognosis and response to chemo- and radiotherapy in several types of cancer. In this mini-review, we discuss the physiological functions of CD98hc, its role in regulating tumor stemness, metastases, and therapy resistance, and the clinical significance of CD98hc as a tumor marker and therapeutic target.

## 1 Introduction

CD98 heavy chain (CD98hc, or 4F2, 4F2HC, 4T2HC, CD98, CD98HC, MDU1, NACAE) is a type II transmembrane glycoprotein identified in activated lymphocytes (1). It is encoded by the solute carrier family 3 member 2 (SLC3A2) gene in humans. The gene is mapped to the 11q12.3 chromosomal region, and encodes 4 transcript splice variants (according to the RefSeq database). Full-length CD98hc is a glycosylated type II transmembrane protein consisting of 630 amino acid residues (as for the transcript variant 3, NM_002394) and composed of four structural regions: intracellular N-tail (100 - 184 amino acid residues), single transmembrane domain (mapped within 185-205 amino acid residues), domain linker and glycosylated extracellular domain including 206-630 amino acid residues (2). CD98hc serves as a chaperone for six amino acid transporters, providing their recruitment to the plasma membrane. In particular, CD98hc binds with one of the light chains (L-type amino acid transporter 1 (LAT1), LAT2, y^+^LAT1, y^+^LAT2, cystine/glutamate antiporter (xCT) and Asc-type amino acid transporter 1 (Asc1) through disulfide bond and electrostatic interactions to form CD98 protein (2–5). In addition, CD98hc modulates intracellular signaling by its direct physical association with cell adhesion receptor integrin β1 (6), or glycoprotein CD147, an essential regulator of lactate and pyruvate transport (7), and regulates glucose uptake (8) that makes CD98hc a multifunctional hub protein. Indeed, lack of CD98hc triggers amino acid and glucose uptake inhibition, glycolysis suppression, decrease in the intracellular levels of nucleotides through the defective pentose phosphate pathway (PPP), oxidative stress, and cell cycle arrest (9–11). Overexpression of CD98hc drives malignant transformation (12, 13) and is associated with progression in different human malignancies. CD98hc and CD98hc binding partners are critical in regulating cancer cell functional properties (**Table 1**). A high expression of CD98hc has been related to the histopathological features and clinical prognosis in patients with many solid cancers, such as head and neck squamous cell carcinoma (HNSCC), glioma, colon adenocarcinoma, pancreatic ductal adenocarcinoma (PDAC), non-small cell lung cancer (NSCLC) and breast cancer (18, 35–41), and tumor response to conventional therapies, such as chemo- and radiotherapy (11, 42, 43). Thus, CD98hc plays a vital role in both physiological and pathological conditions. This mini-review discusses the role of CD98hc in regulating tumor growth, metastasis, and therapy response. We also highlight the clinical significance of CD98hc as a marker of tumor progression and therapy resistance and a promising therapeutic target to enhance the efficacy of conventional anti-cancer therapy.

**Table 1:** The exemplary studies of the roles of CD98hc and CD98hc binding partners in regulating the cancer cell functional properties.

Gene & protein ID	Functions	Tumor entity	Analysis	References
SLC3A2 (CD98hc)	Cell proliferation, viability, clonogenicity and cell cycle	RCC	Gene silencing, [^3^H] thymidine incorporation	(14)
		HNSCC	Gene silencing, cell viability assay, colony formation assay	(11, 15)
		Osteosarcoma	Gene silencing, cell viability assay, cell cycle flow cytometry analysis, colony formation assay	(16)
		Lung adenocarcinoma	Overexpression of the SLC3A2-NRG1 fusion gene, cell viability assay, colony formation assay	(17)
	Tumor growth	RCC	Gene silencing, xenograft murine tumors	(14)
		Skin squamous cell carcinoma (SCC)	Genetically engineered mice *K14-CreERT2, CD98hc^fl/fl^*, chemical skin carcinogenesis	(18)
		Lung cancer	Overexpression of the SLC3A2-NRG1 fusion gene, xenograft murine tumors	(17)
	Migration, invasion	RCC	Gene silencing, cell adhesion and spreading on fibronectin, cell transwell migration	(14)
		Lung cancer	Overexpression of the SLC3A2-NRG1 fusion gene, cell transwell migration	(17)
	Oxidative stress, Ferroptosis	HNSCC	Gene silencing, production of mitochondrial superoxide, lipid ROS analysis, iron measurements	(15)
		HNSCC	Gene silencing, GSH/GSSG ratio, CM-H2DCFDA staining	(11)
		Lung cancer	Gene silencing, lipid peroxidation assay	(19)
		Different cell lines	Gene silencing, cell viability analysis in response to Erastin treatment	(20)
	Autophagy	HNSCC	Gene silencing, Autophagy Green™ staining, Western blotting, PCR analysis of autophagy gene activation	(11)
	Apoptosis	RCC	Gene silencing, Annexin V/PI flow cytometry	(14)
	Radioresistance	HNSCC	Gene silencing, 2D and 3D clonogenic analyses	(11)
SLC7A5 (LAT1)	Cell proliferation, viability, clonogenicity and cell cycle	Different cell lines	Gene silencing, chemical inhibition with JPH203, viable cell counting, spheroid growth inhibition, colony formation assay	(21)
		CRC	Gene silencing, chemical therapy with oxaliplatin, cell viability assay	(22)
		Breast cancer	Chemical inhibition with JPH203, cell viability assay	(23)
		Medulloblastoma	Chemical inhibition with JPH203, viable cell counting, spheroid growth inhibition	(21)
		Lung cancer	Gene silencing, cell viability assays	(24)
		Cholangiocarcinoma	Chemical inhibition with JPH203, cell viability assay, cell cycle flow cytometry analysis	(25)
		T-cell lymphoblastic lymphoma/T-cell acute lymphoblastic leukemia	Chemical inhibition with JPH203, cell viability assays, BrdU incorporation	(26)
	Migration, invasion, metastasis	Lung cancer	Gene silencing, scratch assay	(24)
		Medulloblastoma	Chemical inhibition with JPH203 and scratch assay	(21)
	Tumor growth	Colon cancer	Gene silencing, xenograft murine tumors	(21)
		Cholangiocarcinoma	Chemical inhibition with JPH203, xenograft murine tumors	(25)
		T-cell lymphoblastic lymphoma/T-cell acute lymphoblastic leukemia	Chemical inhibition with JPH203, xenograft murine tumors	(26)
	Apoptosis	Cholangiocarcinoma	Chemical inhibition with JPH203, western blotting for cleaved caspase 3	(25)
		T-cell lymphoblastic lymphoma/T-cell acute lymphoblastic leukemia	Chemical inhibition with JPH203, Annexin V/DAPI flow cytometry	(26)
	Radioresistance	HNSCC	Gene silencing, clonogenic survival assay	(11)
SLC7A11 (xCT)	Proliferation, Cell viability, cell cycle	Prostate cancer	Chemical inhibition with Erastin, colony formation assay	(27)
		RCC	Gene silencing and overexpression, cell viability assay and cell cycle flow cytometry analysis	(28)
		HNSCC	Gene silencing, cell viability assay, colony formation assay	(29)
		Gastric cancer	Gene silencing, viable cell counting	(30)
		Different cell lines	Chemical inhibition with Erastin, multi-cellular tumor spheroid growth inhibition, cell viability assay	(31)
	Migration, invasion, metastasis	Glioblastoma	Chemical inhibition with sulfasalazine and scratch assay	(32)
		Gastric cancer	Gene silencing, cell transwell migration	(30)
		Prostate cancer	Chemical inhibition with Erastin, Matrigel drop invasion assay	(27)
		RCC	Gene silencing and overexpression, cell transwell migration and scratch assay	(28)
		HNSCC	Gene silencing, cell transwell migration and scratch assay	(29)
	Tumor growth	Glioblastoma	Gene silencing and orthotopic tumor growth in xenograft murine models	(32)
		Prostate cancer	Chemical inhibition with Erastin, tumor growth in xenograft murine models	(27)
	Oxidative stress, Ferroptosis	Gastric cancer	Chemical inhibition with Erastin, gene silencing and gene overexpressing, lipid peroxidation assay	(30)
		Prostate cancer	Chemical inhibition with Erastin, CM-H2DCFDA staining	(27)
		Pancreatic ductal adenocarcinoma (PDAC)	Gene silencing in the genetically engineered mice model *Kras^FSF.G12D/+;^ Tp53^R172H/+;^ Pdx1FlpO^tg/+;^ Slc7a11^Fl/Fl^; Rosa26^CreERT2/+^*	(33)
		Melanoma	Gene silencing with or without SLC7A11 overexpression, tumor radiosensitization in vitro and in xenograft murine models by inducing lipid ROS production	(34)
	Apoptosis	HNSCC	Gene silencing, Annexin V/PI flow cytometry	(29)
	Radioresistance	Fibrosarcoma	Gene silencing, clonogenic survival assay	(34)

RCC, renal cell carcinoma; HNSCC, head and neck squamous cell carcinoma; CM-H2DCFDA, 5-(and-6) chloromethyl-2′,7′ dichlorodihydrofluorescein diacetate acetyl ester; CRC, colorectal cancer; NRG1, neuregulin 1; PI, propidium iodide.

## 2 Biological functions and signaling mechanisms mediated by CD98hc

Targeted disruption of the CD98hc gene and analysis of the chimeric mice models demonstrated that CD98hc contributes to embryogenesis. During the early stage of embryonic development, embryos that lack CD98hc die shortly after implantation due to defective integrin signaling (44, 45), whereas the amino acid transport regulated by CD98hc becoming indispensable for the embryonic development at later stages (44). Embryonic stem (ES) cell lines without CD98hc expression showed a low ability to spread on fibronectin (FN) or laminin. CD98 expression enables FN matrix assembly through CD98hc–integrin interaction and consequent activation of RhoA-mediating extracellular matrix contraction (46).

**Figure 1:**
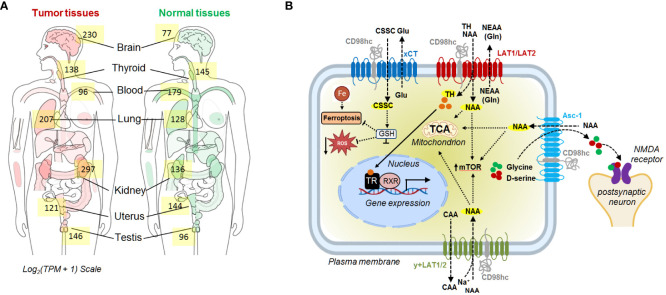
**(A)** The median expression of SLC3A2 in tumor and normal tissue. The data are obtained using GEPIA 2 (49). **(B)** Schematic representation of the CD98hc-related transport systems. CAA, cationic amino acids; CSSC, cystine; Gln, glutamine; Glu, glutamate; GSH, glutathione; NAA, neutral amino acids; NEAA, non-essential amino acids; NMDA, N-methyl-D-aspartate receptor; TCA, tricarboxylic acid cycle; TH, thyroid hormones; TR, thyroid hormone receptors; RXR, retinoid X receptor.

CD98hc is expressed in all human organs, especially in placenta, bone marrow, kidney, lung, uterus, and thyroid tissues (47, 48) (**Figure 1A**), and is essential for the proliferation, survival, and functioning of different types of normal cells, including vascular smooth muscle cells (50), central nervous system (51), skin (52), dental cells (53), and other tissues (https://www.ncbi.nlm.nih.gov/gene/6520). The reduction of CD98hc expression resembles age-related skin alterations in mice models with tamoxifen-inducible epidermis-specific CD98hc knockout. CD98hc deletion in basal keratinocytes inhibits epidermal wound healing and hair growth due to deficient epidermis regeneration by self-renewal, keratinocyte proliferation, and migration. These effects are mediated by defective c-Src/focal adhesion kinase (FAK) signaling, persistent RhoA activation, and reactive oxygen species (ROS) accumulation due to insufficient amino acid (AA) availability (52). Indeed, two core functions of CD98 in different types of cells are to assist in transporting AA into and out of cells and regulate downstream signaling pathways, including extracellular regulated protein kinase (ERK), phosphatidylinositol-3-kinase (PI3K), FAK, Src, and Rho GTP, through its binding to the interacting partners, such as CD147 or integrins (54, 55). CD98hc forms a complex with integrin β1 or β3 subunits, and promotes anchorage independent cell growth and integrin-mediated cell signaling such as phospho-FAK, Akt and mitogen-activated protein kinase (MAPK)/extracellular signal-regulated kinase (ERK) pathways (14, 56, 57). The interaction between CD98hc and integrins is important for the regulation of stemness, proliferation, cell survival, and cancer transformation (14, 56, 57).

**Figure 2:**
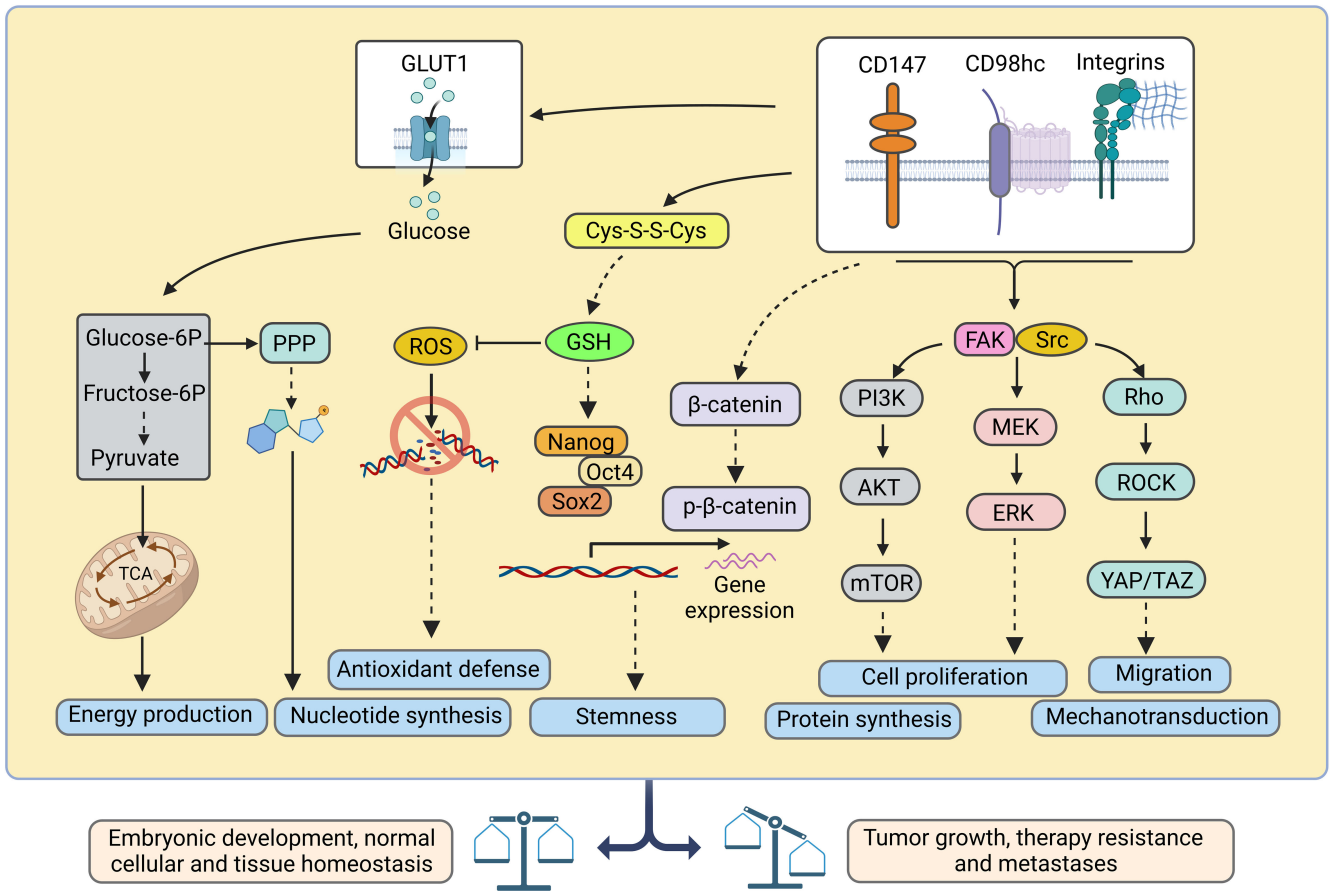
Ideograph of downstream pathways regulated by CD98hc and its partners. CD98hc-dependent pathways are critical for embryonic development and normal cellular and tissue homeostasis, and their deregulation is associated with tumor growth, therapy resistance, and metastases. CD98hc, CD98 heavy chain; Cys-S-S-Cys, cystine; FAK, focal adhesion kinase; ERK, extracellular signal-regulated kinase; GLUT1, glucose transporter type 1; GSH, reduced glutathione; MEK, mitogen-activated protein kinase kinase; mTOR, mammalian target of rapamycin; PI3K, phosphoinositide 3-kinases; PPP, pentose phosphate pathway; ROCK, Rho-associated protein kinase; ROS, reactive oxygen species; TAZ, transcriptional coactivator with PDZ-binding motif; YAP, yes-associated protein 1. Created with BioRender.com.

Heterodimerization of CD98hc with light subunits of the SLC7 family is essential for their trafficking, membrane topology, stability, and transport activity (2, 58, 59), although CD98hc does not directly contribute to the amino acid transport (2). CD98hc-LAT1 (SLC7A5) and LAT2 (SLC7A8) are responsible for the transport of neutral amino acids, such as tyrosine, phenylalanine, leucine, cysteine, isoleucine, methionine, valine, tryptophan, as well as histidine. (5, 60, 61). In addition, recent studies using cryo–electron microscopy (cryo-EM) shed light on the structural features defining the specificity of LAT2 toward small neutral amino acids and glutamine (5). Another study based on the analysis of the cryo-EM structure of CD98hc-LAT1 heterodimer revealed the presence of four N-glycosylated Asn residues within the extracellular CD98hc domain and confirmed that this glycosylation is not directly involved in the formation of the CD98hc-LAT1 complex (2). Instead, the recent finding suggested the role of this glycosylation in regulating CD98hc stability and trafficking to the plasma membrane and, consequently, LAT1 intracellular distribution and function (62). In agreement with this observation, other studies also demonstrated that knockout of CD98hc expression resulted in the cytoplasmic localization of LAT1 (11, 63). CD98hc-LAT1 and CD98hc-LAT2 mediated amino acid transport is necessary to meet the energetic and nutritional demands, as evidenced by low protein synthesis and proliferation rate in CD98hc knockout cells (10). Furthermore, CD98hc-LAT1 and CD98hc-LAT2 mediate the transport of the amino acid-derived thyroid hormones, mainly 3,3'--diiodothyronine (3,3'-T2) (64), essential for energy metabolism and developmental processes (65, 66). CD98hc-y^+^LAT1 (SLC7A7) and CD98hc-y^+^LAT2 (SLC7A6) heterodimers play a role in the efflux of cationic amino acids, such as arginine, lysine, and ornithine in exchange for neutral amino acids and Na^+^ (67, 68). CD98hc-xCT (SLC7A11) functions as a cystine/glutamate antiporter, which transports cystine into the cell in exchange for glutamate (69). Cystine plays a critical role in cellular antioxidant defense and redox balance by influencing the availability of cysteine, which is a precursor for the synthesis of glutathione (GSH) (70). GSH, as the primary intracellular antioxidant, contributes to neutralizing ROS and protects cells from oxidative damage (70, 71). CD98hc-Asc1 (SLC7A10) regulates the D-serine and glycine transport in the central nervous system. Both D-serine and glycine serve as co-agonists of the N-methyl-D-aspartate (NMDA) receptor (72). Finally, CD98hc is reported to bind GLUT1 and prevent its lysosomal degradation, thereby increasing glucose uptake (8) (**Figure 1B**). Furthermore, studies using mass-spectrometry based identification of the protein-protein interaction demonstrated that CD98hc interacts with PTPRJ, a receptor protein tyrosine phosphatase regulating CD98hc proteasomal degradation (73), and ASCT2 (SLC1A5) transporter mediating glutamine uptake (74). The mass spectrometry analysis suggested that CD98hc, CD147, monocarboxylate transporters (MCTs), and ASCT2 are part of the cell surface protein complex regulating cell energy metabolism and biosynthesis by the coordinated transport of amino acid and lactate (74). CD147 mediates the membrane localization of CD98hc and the activation of the downstream signaling mechanisms (75). CD147-CD98hc complex regulates various cell functions contributing to cell proliferation (55), metabolism (74), drug resistance (76), and cell aggregation (77) and leading to the activation of several key signaling pathways such as β-catenin (78), β1-integrin (75) and PI3K/AKT (55, 75) (**Figure 2**).

## 3 CD98hc as a regulation of immunity

CD98hc is an essential regulator of innate immunity and adaptive immune responses, mediating the functions of T and B lymphocytes and macrophages. In 1981, CD98hc was discovered as a cell surface marker present on the activated human lymphocytes and monocytes (1). The following findings revealed that CD98hc regulates B cell proliferation, spreading, and formation of the antibody-producing plasma cells through the integrin and MAPK/ERK/p27 signaling mechanism (79). Furthermore, the same team later demonstrated that the interaction of CD98hc with integrins regulates T cell proliferation (80), whereas CD98hc loss is associated with impaired antigen-driven T cell clonal expansion *in vivo* and prevents autoimmune response in murine modes of type I diabetes (80). Interestingly, inhibiting CD98hc on monocytes using anti-CD98 monoclonal antibody was also shown to suppress T cell proliferation (81). Anti-CD98hc antibody was suggested as a possible approach to increase transplantation efficacy since CD98hc deletion in T cells was associated with attenuated lymphocyte migration, low proliferation in response to the alloantigens, and poor allograft infiltration (82). On the other hand, the presence of CD98hc is critical for the control of immune tolerance. Blocking CD98hc and CD147 interaction leads to the degradation of the Foxp3 protein, one of the key transcription factors driving the differentiation and functions of regulatory T cells (Treg). Consequently, Treg cell stability requires cell-cell contact associated with CD98hc-CD147 interaction, sequestering of cyclin-dependent kinase 2 (CDK2) from activation and Foxp3 stabilization (83). Conditional deletion of CD98hc inhibits antigen-presenting and phagocytic activities of macrophages and is associated with decreased p130cas phosphorylation and impaired activation of AKT, ERK, and c-Jun N-terminal kinase (JNK) after treatment with macrophage colony-stimulating factor (M-CSF) and receptor activator of nuclear factor κB ligand (RANKL), the inducers of macrophage differentiation into osteoclasts. As a result, osteoclast formation by peritoneal macrophages isolated from CD98hc-defect mice was severely impaired (84). Nevertheless, CD98hc knockout mice have normal trabecular bones, and the function of CD98hc for *in vivo* osteoclast formation still needs to be clarified (84). CD98hc and its binding partners integrin β1 and CD147 regulate the actin cytoskeleton and affect monocyte adhesion by activating integrin-mediated signaling (85). CD98hc also serves as a surface receptor to mediate the internalization and trafficking of β-defensin 3 (hBD3), a peptide regulating innate immune response (86). This finding could potentially suggest that CD98hc/hBD2 interplay can play a role in the innate immune surveillance of tumor cells (86, 87).

## 4 CD98hc as a pathogen entry protein

In 1992, CD98hc was described as a fusion regulatory protein (FRP)-1 (gp80) regulating cell fusion upon Newcastle disease virus (NDV) infection (88). Since then, CD98hc has been shown to mediate many host-pathogen interactions (89). In particular, CD98hc provides an entry by endocytosis for the vaccinia virus (VV) in different *in vitro* models, including mouse embryonic fibroblasts (MEF) and human HeLa cells (90). CD98hc and VV particles co-localize in plasma membrane lipid rafts of the host cells upon infection, and genetic silencing of CD98hc expression reduced virus entry (90). CD98hc was identified as one of the proteins interacting with mouse norovirus-1 (MNV-1). The infection of mouse macrophages by MNV-1 is reduced after CD98hc depletion (91). *Plasmodium vivax* is a pathogenic protozoal parasite causing human malaria through the invasion of reticulocytes (92). *Plasmodium vivax* reticulocyte binding proteins (PvRBP) serve as invasion ligands mediating reticulocyte invasion (92). A recent study identified CD98hc as a reticulocyte-specific receptor for PvRBP2a, providing an additional route for *Plasmodium vivax* infection (93). Upon Herpes simplex virus 1 (HSV-1) infection, CD98hc and β1 integrin interact with HSV-1 proteins and mediate nucleocytoplasmic transport of perinuclear virions to release viral nucleocapsids into the cytosol (94, 95). CD98hc is also suggested to regulate viral gene expression upon Kaposi’s sarcoma-associated herpesvirus (KSHV) infection (96). Furthermore, CD98hc plays a role in bacterial infection. In particular, CD98hc has been identified as a binding partner for the VirB2 pilus protein of gram-negative coccobacilli *Brucella* and is essential for bacterial uptake and intracellular replication (97).

## 5 CD98hc as a promoter of tumor growth and metastases

**Figure 3:**
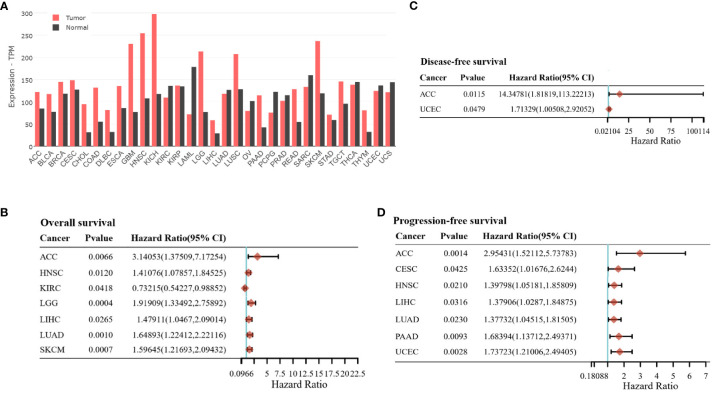
**(A)** The SLC3A2 gene expression in tumor samples and normal tissues. The data are obtained using GEPIA 2 (49) based on The Cancer Genome Atlas (TCGA) and the Genotype-Tissue Expression (GTEx) datasets. TCGA Study Abbreviations: LAML, Acute Myeloid Leukemia; ACC, Adrenocortical carcinoma; BLCA, Bladder Urothelial Carcinoma; LGG, Brain Lower Grade Glioma; BRCA, Breast invasive carcinoma; CESC, Cervical squamous cell carcinoma and endocervical adenocarcinoma; CHOL, Cholangiocarcinoma; COAD, Colon adenocarcinoma; ESCA:Esophageal carcinoma; GBM:Glioblastoma multiforme; HNSC, Head and Neck squamous cell carcinoma; KICH, Kidney Chromophobe; KIRC:Kidney renal clear cell carcinoma; KIRP:Kidney renal papillary cell carcinoma; LIHC, Liver hepatocellular carcinoma; LUAD:Lung adenocarcinoma; LUSC:Lung squamous cell carcinoma; DLBC, Lymphoid Neoplasm Diffuse Large B-cell Lymphoma; OV, Ovarian serous cystadenocarcinoma; PAAD, Pancreatic adenocarcinoma; PCPG, Pheochromocytoma and Paraganglioma; PRAD, Prostate adenocarcinoma; READ:Rectum adenocarcinoma; SARC:Sarcoma; SKCM, Skin Cutaneous Melanoma; STAD, Stomach adenocarcinoma; TGCT:Testicular Germ Cell Tumors; THYM, Thymoma; THCA, Thyroid carcinoma; UCEC, Uterine Corpus Endometrial Carcinoma; TPM, transcripts per million. **(B, C)** The association between SLC3A2 gene expression and prognosis profile, including overall survival **(B)**, disease-free survival **(C)**, and progression-free survival **(D)** across different tumor entities. The data are obtained using the GSCALite platform (98) based on The Cancer Genome Atlas (TCGA) data.

The high expression levels of CD98hc in tumors compared to normal tissues could serve as indirect evidence for the contribution of CD98hc in tumor development. Pan-cancer analysis showed that CD98hc is highly expressed in many cancer types, including HNSCC, glioblastoma, colon, lung, kidney, pancreatic cancer and melanoma (**Figure 3A**). Indeed, there is a growing research interest in the and melanoma oncogenic roles of CD98hc. Lose and gain function studies showed CD98hc appears essential for tumor initiation, progression, and metastatic development (12, 13, 18, 76, 99–104). Overexpression of CD98hc in fibroblasts inhibits apoptosis, drives malignant transformation, and induces tumor formation in immunodeficient mice (12, 13, 105). CD98hc boosts cancer cell proliferation and tumor growth by transporting amino acids (10, 11), augmenting the integrin signaling (18, 54), and activation of the mTOR (11, 15, 17), PI3K/AKT (16) and MAPK signaling pathways (104, 106). CD98hc is essential for sustaining glucose uptake, glycolysis, and pentose phosphate pathway (PPP) (8, 10), fueling the Krebs cycle (11) and therefore maintaining cell energy metabolism. CD98hc also promotes the cell cycle by providing raw materials for nucleotide synthesis (10).

Furthermore, CD98hc is essential for sensing the extracellular matrix (ECM) stiffness and tumor cell migration and invasion. The ECM is made up of fibrous proteins such as collagens, elastin, and glycoproteins, including fibronectin, proteoglycans, and laminin (107). ECM is dynamically regulated in normal and disease conditions (108). Exposure of human bronchial epithelial cells to the diesel exhaust particle (DEP) extract induced expression of CD98hc and upregulation of mRNA levels of matrix metalloproteinase (MMP)-2 (109), a zinc-dependent endopeptidase mediating the degradation of ECM components and tumor cell invasion (110, 111). Consistently, silencing of CD98hc reduced the levels of MMP2 (109). Integrins mediate the communication between ECM and cancer cells through binding to ECM proteins, such as FN and vitronectin (VN) (112). CD98hc promotes tumorigenesis by interacting with integrins β1 and β3 and activating adhesive signals, such as focal adhesion kinase (FAK) regulating actin cytoskeleton dynamics. Acquisition of the aggressive tumor characteristics in clear cell renal cancer (ccRCC) cells is attributed to the CD98hc-integrin binding, whereas silencing of CD98hc decreases tumor cell spreading, migration and proliferation, and tumor growth *in vivo* (14). CD98 overexpression in the intestinal epithelial cell is associated with activation of the MEK/ERK signaling, increases levels of proinflammatory cytokines, and leads to tumorigenesis (113). Furthermore, a recent study demonstrated that CD98hc promotes colon cancer metastases through the crosstalk of tumor cells and tumor-associated neutrophils (TANs) (106). In particular, colorectal cancer cells secret transforming growth factor beta1 (TGF-β1) to induce neutrophils to become anterior gradient-2 (AGR2) positive TANs (92). TANs secrete AGR2 in the tumor microenvironment. CD98hc, expressed in colorectal cancer (CRC) cells, is the functional receptor for secreted AGR2 and physically interacts with AGR2 via its extracellular region. CD98hc-AGR2 binding leads to increased xCT activity and intracellular level of reduced GSH and promotes CRC liver metastasis through MAPK/ERK and RhoA/Rho-associated protein kinase 2 (ROCK2) pathway (106). The high levels of CD98hc expression in metastatic tissues compared to primary tumors have been found in different types of solid cancers (114, 115), and CD98hc expression in primary tumors is associated with increased metastatic development in HNSCC (43, 116), breast cancer (36), and gastric cancer (117). CD98hc mediates integrin-driven mechanotransduction. Furthermore, CD98hc increases the stiffness of the tumor microenvironment by activation of the actin-Rho/Rho-associated protein kinase (ROCK) and YAP/TAZ signaling, resulting in changes in collagen and fibronectin organization (18). Integrin-mediated mechanotransduction plays a role in Ras-induced development of skin squamous cell carcinoma, and conditional knockout of CD98hc expression in epidermis inhibited chemical skin carcinogenesis mediated by 7,12-dimethylbenz(a)anthracene (DMBA)-induced oncogenic Ras mutations. These findings suggest that CD98hc mediates Ras-induced tumor growth (18). A fusion of CD98hc with other oncogenes affects its functions and pathophysiological role. More than 20% of patients with invasive mucinous adenocarcinoma of the lung were found to have a fusion of CD98hc and a driver oncogene protein Neuregulin 1 (NRG1). CD98hc-NRG1 fusion promotes the proliferation, invasion, migration, and tumor growth of lung cancer cells via the upregulation of PI3K/AKT/mTOR and FAK-Src pathways (97). Furthermore, patients with CD98hc-NRG1 fusions showed significantly lower overall survival and disease-free survival compared to those without gene fusion (17). All these findings indicate that CD98hc regulates cancer cell proliferation and migration via modeling the tumor microenvironment and directly activating the signaling cascades in tumor cells.

One of the tumor hallmarks is metabolic rewiring induced by oncogenic mutations and epigenetic changes. This metabolic reprogramming is often associated with upregulation of ROS levels. Amino acid uptake mediated by CD98hc plays a role in tumor cell protection against oxidative stress (71). In particular, CD98hc is essential to prevent ferroptosis, a form of regulated cell death characterized by the accumulation of lipid peroxides and iron-dependent ROS in cells (118). It is emerging as an essential process in cancer biology, with implications for both cancer development and therapy (31). For example, one of the promising anti-cancer therapeutic strategies via ferroptosis induction is the inhibition of glutathione peroxidase 4 (GPX4), an enzyme catalyzing the peroxide reduction at the expense of GSH and therefore preventing cells from ferroptosis (119). Cystine transporter system Xc^-^ composed of catalytic subunit xCT/SLC7A11 and chaperone subunit CD98hc transfers extracellular cystine into cells and converts it into cysteine, which is then used to synthesize GSH, a crucial ROS scavenger and cofactor of GPX4 to reduce peroxides to corresponding alcohol molecules (120). As a key component of system Xc^-^, CD98hc has been shown to be a ferroptosis mediator in HNSCC, lung, and prostate cancer (15, 19, 27). Of interest, exosomes of the hepatitis B virus (HBV)-positive hepatocellular carcinoma (HCC) cells induce ferroptosis in tumor-suppressing M1-type macrophages by inhibiting CD98hc expression through exosomal miR-142-3p (121). This finding can explain a depletion of M1-type macrophages in HBV^+^ HCC tissues (121). Inhibition or downregulation of SLC7A11 (xCT) leads to reduced cystine uptake, resulting in low intracellular GSH levels, accumulation of ROS, and increased susceptibility to ferroptosis (122). Blocking SLC7A11 (xCT) by a small molecule compound, erastin, induces ferroptosis in cancer cells (27, 30, 123). All this evidence indicates that CD98hc and CD98hc-related amino acid transporters play a vital role in fast-growing tumor cells by regulating energy metabolism, biosynthesis, and key oncogenic pathways.

## 6 CD98hc and CD98hc-related amino acid transporters as potential markers and regulators of cancer stem cells

Cancer stem cells (CSCs) are a population of cancer cells defined by their ability to self-renew and differentiate. CSCs give rise to tumorigenic and non-tumorigenic cell populations and maintain tumor growth (124–126). Cancer cell plasticity represents a significant challenge for target CSCs (127). However, if CSCs are not eradicated during the course of treatment along with non-CSC populations, they might lead to tumor recurrence (128). The subpopulations of CSCs are responsible for tumor cell dissemination and metastatic growth (129). Some CSC populations are also proven to be resistant to the conventional treatment (128, 130, 131). CSC markers were related to the poor clinical prognosis in different types of cancer (124, 128, 132, 133). CD98hc and CD98hc-binding proteins, such as SLC7A5 and SLC7A11, have been characterized as putative CSC markers for several malignancies (134–138) (**Table 2**). These studies mainly used spherogenicity assays *in vitro* and CSC-related gene expression analyses to assess the CSC phenotypes, and only some of them employed tumor transplantation assay, a “gold standard” approach for characterizing CSC populations (142). In particular, Martens-de Kemp et al. used limiting dilution analysis and serial transplantation of CD98^high^ and CD98^low^ HNSCC cells to demonstrate that CD98^high^ cells are self-renewing population, and CD98^high^ cell–derived tumors histologically recapitulate the parental tumor tissues (135). Bajaj et al. demonstrated that CD98hc promotes acute myelogenous leukemia (AML) propagation in mice models of disease by maintaining leukemic stem cells through the integrin signaling pathway, and genetic loss of CD98hc or antibody-mediated CD98hc blockage impairs *in vivo* AML propagation (56). CD98hc increases the incidences of intestinal tumors in mice bearing a mutation in Apc tumor suppressor (113) and in a murine model of colitis-associated CRC (102). On the other hand, the study by Huang et al. based on the transcriptome analysis suggested that CD98hc suppresses CSCs in human cervical carcinoma HeLa cells grown in 3D culture (143). The role of xCT/SLC7A11 in regulating CSCs and its potential targeting is also an actively investigated topic. Immunotargeting of xCT using DNA-based vaccination of mice bearing syngeneic breast tumors inhibits the growth of primary tumors and prevents metastasis formation (138). It was suggested that xCT plays a role in the maintenance of breast CSC cells by regulating GSH production and intracellular redox balance (138). Breast CSCs were found to secrete DKK1 (Dickkopf WNT Signaling Pathway Inhibitor 1), which activates xCT expression in the metastatic cells, protecting them from ferroptosis and increasing metastatic colonization in murine models (139). Similarly, inhibition of xCT by chemical inhibitor erastin targets colorectal CSCs *in vitro* and *in vivo* and attenuates their chemoresistance by elevating ROS levels and inducing ferroptosis (140). Stem cell surface marker CD44v6 induces GSH synthesis by stabilizing the xCT expression, thereby reducing the ROS level and promoting drug resistance of cancer cells (144). In triple-negative breast cancer, chemotherapy induces xCT expression and GSH synthesis in a hypoxia-inducible factor (HIF-1)–dependent manner. Increased levels of intracellular GSH induce nuclear translocation of FoxO3a transcription factor and trigger expression of pluripotency factor Nanog. Consequently, Nanog regulates the expression of other pluripotency factors, such as Oct4 and Sox2, and drives tumor cell reprogramming and CSC enrichment (145). Of interest, the Sox2 transcription factor directly binds to the xCT gene promoter, and Sox2-mediated xCT upregulation protects lung CSCs from ferroptosis (146). Inhibition of xCT with chemical drug sulfasalazine induces apoptosis in CD44v-positive HNSCC cells in murine xenograft models and sensitizes tumor cells to the epidermal growth factor receptor (EGFR)-targeted therapy with cetuximab (147). Thus, based on the current literature (**Table 2**), CD98hc and related amino acid transporters are potential markers for CSCs. Nevertheless, more evidence from the preclinical animal models and patient-derived tissues is warranted to prove that CD98hc and CD98hc-related amino acid transporters serve as markers and regulators of CSCs in different tumor entities.

**Table 2:** CD98hc and its binding partners as cancer stem cell (CSC) markers and regulators (exemplary *in vivo* studies).

Gene	Tumor entity	CSC analyses and models	References
SLC3A2 (CD98hc)	HNSCC	Limiting dilution and serial transplantation assays in nude mice using subcutaneous injection of VU-SCC-OE cells (CD98^high^ and CD98^low^ populations)	(135)
	AML	Establishment of *Cd98hc^fl/fl^;Rosa26-CreER^+/+^* murine AML cells where CD98 is lost after tamoxifen administration and transplantation of these cells into congenic recipient mice; CD98 loss led to a significant increase in survival of mice transplanted with cKit^+^ AML stem cells	(56)
	Intestinal tumors	CD98 overexpression in *Apc^Min/+^* mice resulted in an increase in the incidence of small intestinal and colonic tumors	(113)
	CRC	CD98 overexpression in intestinal epithelial cells in transgenic mice increases colorectal tumorigenesis after treatment with procarcinogen azoxymethane (AOM), followed by induction of chronic colitis by treatment with dextran sodium sulfate (DSS)	(102)
SLC7A11 (xCT)	Breast cancer	Immunotargeting of xCT using DNA-based vaccination of mice bearing syngeneic breast tumors for inhibiting tumor growth and metastases	(138)
		Breast cancer stem cells secretomics; identifying DKK1 as a CSC-secreted protein inducing SLC7A11 expression; a combination of erastin (xCT inhibitor) and gallocyanine (DKK1 inhibitor) for inhibiting experimental breast cancer metastases of MDA-MB-231cells xenografted in nude mice	(139)
	CRC	HT-29 cells, *in vivo* treatment with xCT inhibitor erastin, *in vivo* limiting dilution analysis in nude mice	(140)
SLC7A5 (LAT1)	Neuroblastoma	SLC7A5 knockdown or overexpression in BE-2C and SK-N-SH cells, xenograft tumor growth in nude mice	(141)
	Glioblastoma	U87 and U251 cells, *in vitro* limiting dilution analysis of LAT1^+^ and LAT1^-^ cells and xenograft tumor growth in nude mice	(136)

AML, acure myelogenous leukemia; HNSCC, head and neck squamous cell carcinoma; DKK1, Dickkopf WNT Signaling Pathway Inhibitor 1.

## 7 Clinical significance of CD98hc and CD98hc-associated proteins as potential prognostic markers and therapeutic targets

CD98hc as an oncogene has been correlated with the poor clinical prognosis of patients with different types of cancer (37, 38, 117, 148–154). Elevated CD98hc expression was identified as a prognostic marker for predicting a worse prognosis in patients with pulmonary pleomorphic carcinoma (PPC) (148), biliary tract cancer (149), gastric cancer (117), breast cancer (151), CRC (154), HNSCC (37, 152, 153) and pancreatic cancer (39) (**Table 3**). Our previous study also showed that high expression of CD98hc and LAT1 are associated with poor prognoses in patients with HNSCC treated with primary radiochemotherapy (RCTx) or postoperative radiochemotherapy (PORT-C) (11, 43, 116). On the contrary, low CD98hc expression is an independent factor for predicting poor overall survival (OS) and progression-free survival (PFS) for patients with cutaneous angiosarcoma (CA) (156), although the small sample size is a limitation of this study since CA is a rare malignant tumor. Pan-cancer analysis revealed that CD98hc expression is an independent hazard factor for most cancer types (**Figures 3B–D**).

**Table 3:** Correlation of CD98hc expression with clinical outcomes in patients with malignant diseases (exemplary studies).

Tumor entity	Analysis	Treatment	Patient number	Significant association with clinical endpoints and additional parameters	References
NSCLC with resectable N1 and N2 LN metastases	IHC	SURG, SURG +RT, SURG +CT	220	Postoperative survival	(38)
NSCLC	IHC	SURG	241	DFS; OS; Co-expression with CD147; TNM stage; tumor diameter	(55)
HPV-positive OPSCC	IHC	SURG + RT, RT, CRT, RT + LND + RT (brachytherapy)	711	OS; PFS	(37)
HNSCC	IHC	PORT-C, RCTx	197	LRC; LAT1 expression	(11)
	NanoString RNA analyses	RCTx	158	LRC	(43)
HNSCC, HPV16 DNA negative	RT-PCR and NanoString technology	cisplatin-based PORT-C	195	LRC; Distant metastases	(155)
Gastric cancer	IHC	SURG	331	OS; PFS; tumor stage; LN metastasis; vascular invasion	(117)
Breast cancer (TNBC & non-TNBC)	IHC	SURG	78 (TNBC) 202 (non-TNBC)	OS; DFS	(36)
Invasive breast cancer	IHC, DNA/RNA profiling	SURG, CT for ER-negative and LN-positive patients	1858	DFS in TNBC; DFS in ER+ high-proliferation tumors; *SLC7A5* expression; *SLC7A11* expression; *TP53* mutations; ER− and PR− status; High expression in TNBC; high Ki67staining	(151)
CRC	IHC	SURG	147	OS; DFS; T factor (T1-2/T3-4); Lymphatic permeation; Vascular invasion; LAT1 expression; Ki67 staining	(154)
PPC	IHC	SURG	105	OS; DFS; T factor (T1-2/T3-4); Pathological stage: I-II/III-IV; LAT1 expression	(148)

CRC, colorectal cancer; CRT, chemoradiation; CT, chemotherapy; DFS, disease-free survival; ER, estrogen receptor; HNSCC, head and neck squamous cell carcinoma; HPV, human papillomavirus; IHC, immunohistochemistry; LN, lymph nodes; LND, lymph node dissection; LRC, locoregional control; NSCLC, non-small-cell lung cancer; OPSCC, oropharyngeal squamous cell carcinoma; PFS, progression-free survival; PORT-C, postoperative radio(chemo)therapy; PPC, pulmonary pleomorphic carcinoma; PR, progesterone receptor; RCTx, primary radiochemotherapy; RT, radiotherapy; SURG, surgery, TNBC, triple-negative breast cancer; TNM, tumor (T), nodes (N), and metastases (M); OS, overall survival.

CD98hc is a potential target to improve the therapeutic effect of conventional therapies (157–160). We have demonstrated that CD98hc-associated signaling mechanisms, such as mTOR pathway activation, amino acid metabolism, oxidative stress, and DNA repair, play a central role in regulating HNSCC radioresistance (11, 160). Analysis of the expression of geneset associated with T- and B-cell activation in HNSCC revealed that it negatively correlates with SLC3A2 (CD98hc) expression, and therefore, CD98hc could be potentially associated with tumor immune evasion (157). Thus, strategies harnessing the immune system could be critical for the treatment of immunologic “cold” tumors with high CD98hc expression. A recent study from our group confirmed that CD98hc-redirected UniCAR T cells destroy radioresistant HNSCC spheroids (157). Although immune cells, including T cells, also express CD98hc, the expression level is substantially lower than in tumor cells, and the elimination of CD98hc tumor cells occurs before possible UniCAR T cell fratricide (157, 158). Furthermore, CD98hc regulates breast cancer cell sensitivity to anti-estrogen treatment. Co-expression of the CD98hc/LAT1 complex correlates with endocrine therapy resistance in patients with estrogen receptor (ER) positive/human epidermal growth factor receptor-2 (HER2) negative breast cancer. Depletion of both CD98hc and LAT1 mRNA upregulated the sensitivity of breast tumor cells to a selective estrogen receptor modulator tamoxifen (161). A cell polarity protein Scribble (SCRIB) and CD98hc form a quaternary complex with a mammalian homolog of Drosophila protein lethal giant larvae homolog 2 (LLGL2) and LAT1 to promote plasma membrane localization and stabilizing the amino acid transporters and promote cell proliferation and tamoxifen resistance in ER-positive breast cancer cells. Downregulation of CD98hc expression sensitizes tamoxifen-resistant breast cancer cells to tamoxifen under nutrient stress conditions (162). These studies suggest that CD98hc plays an essential role in endocrine therapy resistance.

Although the development of CD98hc as a therapeutic target is mainly at the stage of preclinical laboratory research, accumulating evidence shows the antitumor roles of CD98hc inhibition in a variety of cancer types (144, 145). Antibody-mediated CD98hc blockade deteriorates cell proliferation and tumor growth both *in vitro* and *in vivo* (56, 163, 164). Furthermore, immunotargeting of the CD98hc binding partners LAT-1 and xCT also has strong antitumor effects in the murine tumor models (138, 165). In 1986, Yagita et al. found that anti-CD98hc antibody specific for the extracellular domain inhibits lymphocyte proliferation (166). Later, Hayes et al. used *in vivo* phenotypic screening to identify anti-CD98hc antibodies with the most potent antitumor properties *in vivo* using different xenograft murine models for Burkitt’s lymphoma, leukemia, and patient-derived lung tumors (163). This study demonstrated that IGN523, a humanized monoclonal antibody, possesses a strong antitumor effect in both hematopoietic malignancies and solid tumors. The molecular mechanisms of the CD98hc-mediated antitumor activities include inhibition of the CD98hc-dependent amino acid transport, induction of antibody-dependent cellular cytotoxicity (ADCC), and increase in the lysosomal permeability leading to cell death (163). It has been evaluated in the early-phase clinical trial for acute myeloid leukemia (NCT02040506). In clinical studies, IGN523 was well tolerated and associated with modest antitumor activity as a single agent, whereas complete or partial responses were not observed for the treated individuals (167). Anti-CD98hc therapy can be potentially tested in solid tumors in the future in combination with standard treatment. Phase I study of another anti-CD98hc antibody, KHK2898 (NCT01516645), was carried out in patients with advanced solid tumors who no longer respond to standard therapy. However, no results were yet posted. A large screening of more than 10,000 monoclonal antibodies raised against multiple myeloma (MM) identified R8H283 antibody specific for the glycosylated form of CD98hc. R8H283 antibody does not bind CD98hc protein on normal hematopoietic cells and possesses a strong anti-MM effect *in vitro* and *in vivo*. The molecular mechanism of the R8H283-mediated anti-MM activities was attributed to ADCC and complement-dependent toxicity (164). A study by Tian et al. screened a phage-display library of single-chain variable fragments (scFvs) targeting CD98hc and identified anti-CD98hc antibody with pH-dependent binding and improved antitumor activity and pharmacokinetic properties in the experimental *in vivo* models (168).

**Figure 4:**
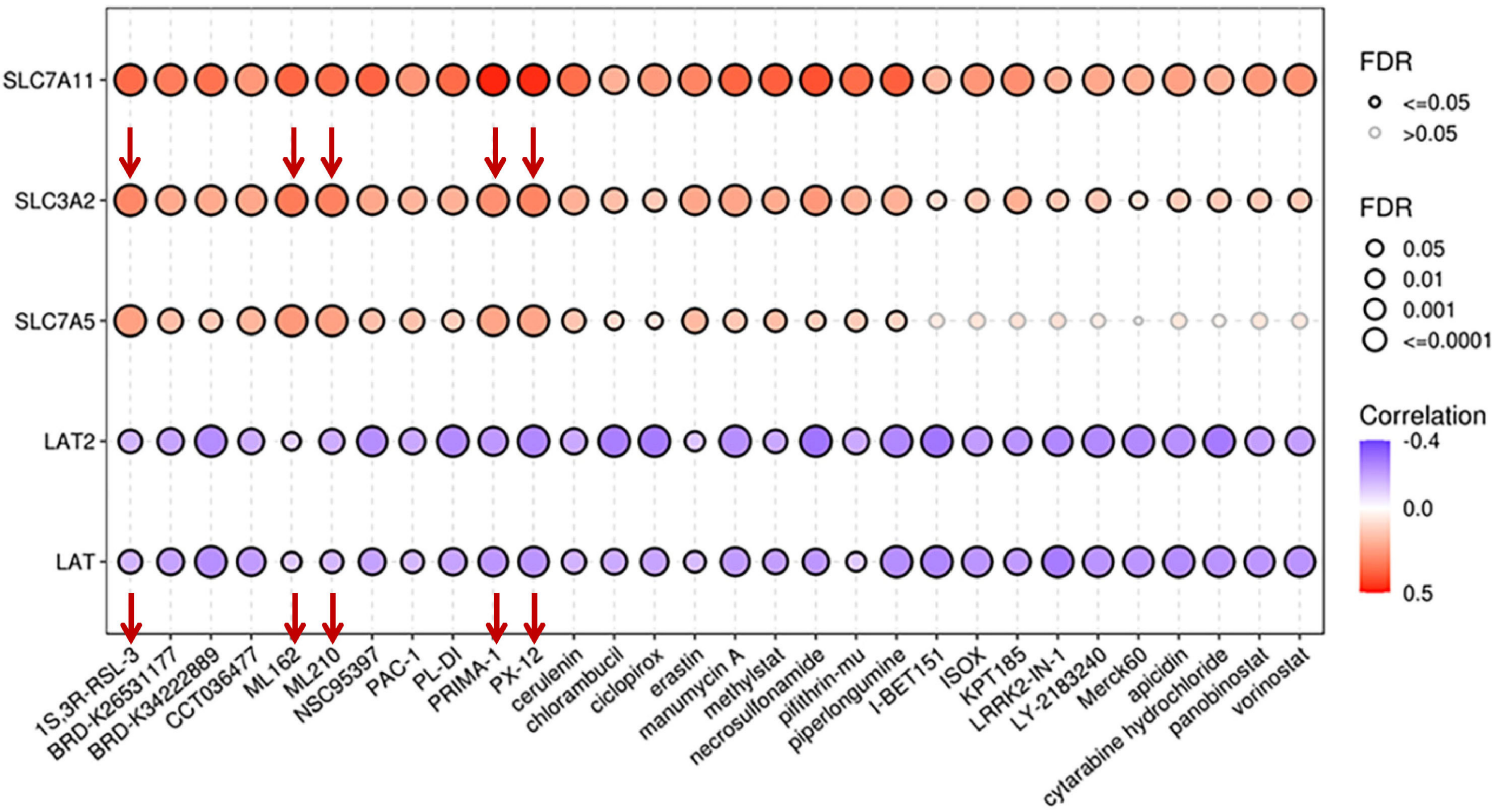
Correlation between drug sensitivity and mRNA expression levels of CD98hc and its binding partners. The data are obtained using the GSCALite platform (98) based on the Cancer Therapeutics Response Portal (CTRP). FDR, false discovery rate.

A Phase I study of JPH203, a LAT1 inhibitor (UMIN000016546), in patients with advanced or refractory solid tumors demonstrated that the drug was well tolerated at low doses and had promising anti-tumor activity in patients with CRC and biliary tract cancer (BTC) (169). A phase II study for patients with advanced BTC (UMIN000034080) will provide more information on JPH203 safety and efficacy. Sulfasalazine is a FDA-approved drug primarily used for the treatment of ulcerative colitis (170) and rheumatoid arthritis (171). It also acts as an xCT inhibitor, reducing cystine uptake and glutathione synthesis (172). Sulfasalazine treatment induces ROS production and synergizes with radiation to increase DNA damage and death of glioblastoma cells *in vitro* (172), inhibits tumor growth in murine models (32), and sensitizes xenograft tumors to radiation (172). Sulfasalazine has been investigated in preclinical and early-phase clinical trials for its safety, drug–drug interactions, and potential anticancer effects for breast and CD44v-positive gastric cancer (173, 174). The treatment of patients with cisplatin refractory gastric cancer with sulfasalazine in combination with cisplatin did not show sufficient antitumor efficacy that could be partially explained by the metabolizing of the orally administered drug in the intestines and, therefore, decreasing its inhibitor potential, suggesting that alternative treatment route should be considered in future studies (174). Another xCT inhibitor, sorafenib, was initially approved by FDA in 2005 for the treatment of advanced renal cell carcinoma (RCC). Since then, it has also been approved for the treatment of hepatocellular carcinoma (HCC) and radio-iodine resistant advanced differentiated thyroid carcinoma (175–177). However, tumors often become drug resistant (178, 179). There are no CD98hc-specific chemical inhibitors available. Based on the results of *in silico* analysis by the Cancer Therapeutics Response Portal (CTRP), CD98hc can be potentially susceptible to some repurposed inhibitors, such as thioredoxin-1 (Trx-1) inhibitor PX-12, GPX-4 inhibitors ML162 and ML-210, inhibitor of GPX-4 and ferroptosis activator (1S,3R)-RSL3, and PRIMA-1 (P53-dependent reactivation and induction of massive apoptosis) compound (**Figure 4**).

Anti-CD98hc radiopharmaceuticals are a promising approach for tumor imaging. Deuschle et al. have developed a high-affinity Anticalin, an engineered protein molecule binding CD98hc with a picomolar affinity for non-invasive biomedical imaging (180). Furthermore, compared with existing tumor-specific positron emission tomography (PET) probes, the LAT1-specific PET probe improved specificity for the early-phase diagnostic application and evaluation of tumor therapy response. LAT1-specific PET probe ^18^F-FIMP has been recently tested in clinics for patients with glioblastoma and demonstrated higher specificity for tumors compared to the ^11^C-MET and [^18^F]Fluorodeoxyglucose (^18^F-FDG) based imaging (181, 182). All this evidence indicates that CD98hc and its binding partners are particularly well-suited targets for diagnostic evaluation and therapeutic intervention in cancers.

## 8 Future perspectives

An accumulating body of evidence has confirmed the contribution of the CD98hc-mediated mechanisms in cancer initiation and progression. The ongoing clinical studies aim to validate the role of CD98hc as a marker of tumor diagnosis and prognosis. Preclinical studies demonstrated that CD98hc is a potent regulator of tumor cell proliferation, invasion, and therapy resistance and a potential target for cancer treatment. Immunotargeting CD98hc demonstrated promising results *in vitro* and *in vivo*. Targeting the CD98hc-binding amino acid transporters is also an additional treatment option, although its antitumor efficacy could be challenged by the previously described transporter plasticity and redundancy (183). Understanding the mechanisms of CD98hc interplay with its partners, such as integrins and CD147, is highly important, and targeting these mechanisms could be another therapeutic option. Although CD98hc-positive cells in HNSCC and oropharyngeal cancer have stem cell properties, it is still a matter of debate whether CD98hc can serve as a marker of CSCs and what its role in the regulation of the tumor self-renewal and differentiation. More evidence from in vivo transplantation assays and patient-derived tumor models would be needed to address these questions. Nevertheless, the levels of CD98hc expression were correlated with clinical outcomes for different tumor entities, suggesting that tumor cells with high CD98hc expression have survival advantages after conventional treatment compared to their counterparts with low CD98hc expression. The ongoing biological studies and further clinical trials for the combination of CD98hc-targeted treatment and conventional therapy, including radiotherapy, might bring breakthroughs in the laboratory research and clinical application of CD98hc in the near future.

## Author contributions

XP and AD wrote, edited and approved the final manuscript. All authors contributed to the article and approved the submitted version.

## Glossary

**Table:**

Asc1	Asc-type amino acid transporter 1
AGR2	anterior gradient-2
ASCT2	alanine serine cysteine transporter 2
CA	cutaneous angiosarcoma
CDK2	cyclin-dependent kinase 2
CSC	cancer stem cell
CRC	colorectal cancer
ECM	extracellular matrix
EpCAM	epithelial cell adhesion molecule
ERK	extracellular regulated protein kinase
ES	embryonic stem cell
FAK	focal adhesion kinase
FN	fibronectin
FRP1	fusion regulation protein 1
JNK	c-Jun N-terminal kinase
GSH	glutathione
GPX4	glutathione peroxidase 4
HBV	hepatitis B virus
HNSCC	head and neck squamous cell carcinoma
HPV	human papillomavirus
KSHV	Kaposi’s sarcoma-associated herpesvirus
LAT1	L-type amino acid transporter 1
MAPK	mitogen-activated protein kinase
M-CSF	macrophage colony-stimulating factor
MCT	monocarboxylate transporter
MEF	mouse embryonic fibroblasts
MMP	matrix metalloproteinases
MNV-1	mouse norovirus-1
NDV	Newcastle disease virus
NRG1	Neuregulin 1
PI3K	phosphatidylinositol-3-kinase
PAT	positron emission tomography
PPP	pentose phosphate pathway
PTPRJ	protein tyrosine phosphatase receptor type J
PvRBP	plasmodium vivax reticulocyte binding protein
RANKL	receptor activator of nuclear factor κB ligand
ROCK	Rho-associated protein kinase
ROS	reactive oxygen species
SLC3A2	solute carrier family 3 member 2
TANs	tumor-associated neutrophils
TFR	transferrin receptor
THs	thyroid hormones
Tregs	regulatory T cells, VV, vaccinia virus
